# Technology development of hyperthermic pressurized intraperitoneal aerosol chemotherapy (hPIPAC)

**DOI:** 10.1007/s00464-021-08567-y

**Published:** 2021-06-10

**Authors:** C. Bachmann, I. Sautkin, G. Nadiradze, R. Archid, F. J. Weinreich, A. Königsrainer, M. A. Reymond

**Affiliations:** 1 Department of General and Transplant Surgery and National Center for Pleura and Peritoneum, University of Tübingen, Tübingen, Germany; 2 NCPP, Universits Hospital Tübingen, Hoppe Seyler Str 3, 72076 Tübingen, Germany

**Keywords:** Intraperitoneal chemotherapy, Hyperthermia, Laparoscopy, Medical devices, Aerosol

## Abstract

**Background:**

Optimized drug delivery systems are needed for intraperitoneal chemotherapy. The aim of this study was to develop a technology for applying pressurized intraperitoneal aerosol chemotherapy (PIPAC) under hyperthermic conditions (hPIPAC).

**Methods:**

This is an ex-vivo study in an inverted bovine urinary bladder (IBUB). Hyperthermia was established using a modified industry-standard device (Humigard). Two entry and one exit ports were placed. Warm-humid CO_2_ was insufflated in the IBUB placed in a normothermic bath to simulate body thermal inertia. The temperature of the aerosol, tissue, and water bath was measured in real-time.

**Results:**

Therapeutic hyperthermia (target tissue temperature 41–43 °C) could be established and maintained over 30 min. In the first phase (insufflation phase), tissue hyperthermia was created by insufflating continuously warm-humid CO_2_. In the second phase (aerosolization phase), chemotherapeutic drugs were heated up and aerosolized into the IBUB. In a third phase (application phase), hyperthermia was maintained within the therapeutic range using an endoscopic infrared heating device. In a fourth phase, the toxic aerosol was discarded using a closed aerosol waste system (CAWS).

**Discussion:**

We introduce a simple and effective technology for hPIPAC. hPIPAC is feasible in an ex-vivo model by using a combination of industry-standard medical devices after modification. Potential pharmacological and biological advantages of hPIPAC over PIPAC should now be evaluated.

**Supplementary Information:**

The online version contains supplementary material available at 10.1007/s00464-021-08567-y.

A cytotoxic effect of hyperthermia was observed as early as the 19th century when patients with malignant tumors experienced a regression of these tumors during high-febrile bacterial diseases [1]. The mechanisms of thermal cell damage have not yet been conclusively elucidated. Cytotoxicity induced by hyperthermia is multifactorial: heat induces changes both in the cell membrane and cell nucleus. Cell metabolism is modified by hyperthermia: many proteins, including Heat-shock proteins, are differentially expressed or functionally modified in hyperthermic cell stress and possibly death [2].

Current hyperthermia strategies generally include local, regional, and whole-body approaches. Several technologies are used, such as microwave, radiofrequency, laser, ultrasound [3] and extracorporeal circulation [4]. For example, intraperitoneal hyperthermic chemotherapy (HIPEC) has been proposed for enhancing the cytotoxic effect of cytostatic drugs on peritoneal malignancies [5]. According to the Einstein–Stokes equation, diffusion of solutes is proportional to temperature, so that drug tissue uptake is expected to increase with temperature. Moreover, hyperthermia increases the permeability of the connective matrix, the transport of large molecules by convection, and this tissue uptake of drugs [6]. Hyperthermia has biological antitumor effects synergistic with chemotherapy [2]. Over the last 30 years, a combination of cytoreductive surgery (CRS) with hyperthermic intraperitoneal chemotherapy (HIPEC) has developed into a standard of care in selected patients with peritoneal surface malignancies [7]. Nevertheless, the potential of hyperthermia to enhance IP drug delivery remains uncertain. Despite hyperthermia, HIPEC still has significant pharmacological limitations, for example, limited drug tissue penetration [8]. In the large animal model, hyperthermia achieved higher concentrations of oxaliplatin in visceral but not parietal surfaces [9]. In the clinical setting, whereas hyperthermia does not seem to increase morbidity and mortality of intraperitoneal chemotherapy [10], there is little clinical evidence on its added efficacy [6]. HIPEC’s clinical efficacy did not always meet the expectations [11, 12].

Twenty years ago, the principle of pressurized intraperitoneal aerosol chemotherapy (PIPAC) was proposed to overcome the pharmacological limitations of HIPEC, such as poor homogeneity of spatial distribution and limited tissue penetration [13]. The rationale of PIPAC is to take advantage of physical laws such as aerosol nature and pressure to optimize drug distribution and tissue uptake. In the meantime, over 12′500 PIPAC procedures have been performed worldwide [14]. In a recent systematic review on 1810 PIPAC in 838 patients. PIPAC was shown to be feasible and safe. Data on objective response and quality of life are encouraging. Therefore, PIPAC can be considered as a treatment option for refractory, isolated peritoneal metastasis of various origins [15].

PIPAC was proposed as a normothermic drug delivery system. A system combining the improved distribution and penetration of drugs using delivery as a pressurized aerosol and the superior antitumor effect of hyperthermia was missing. This concept and the related technology (H-PAC or hPIPAC) was first developed in 2015 by H.H. Kim in South Korea and tested in the large animal model [16]. It was possible to generate and maintain hyperthermia within the lower therapeutic range (38.7–41.0 °C for an extended period (1 h). Widespread staining of indocyanine green was documented throughout the intraperitoneal cavity. Cisplatin was applied at a dose of 25 mg in two animals, a laparoscopic colon resection was performed, and no delay in anastomotic healing was observed. Thus, hPIPAC is feasible and might represent significant progress in treating peritoneal metastasis.

However, the technology proposed appears to have some limitations. First, the heating apparatus is complex and voluminous, making it challenging to implement in a standard operating room. Second, the system needs further development to allow proper sterilization. Finally, obtaining regulatory approval for such a complex system might be challenging. Against this framework, we noted an existing, certified technology (Humigard^®^, Fischer & Paykel Healthcare, Auckland, New Zealand) for heating and humidifying CO_2_. Therefore, we decided to investigate the potential of this technology for generating hPIPAC.

In this paper, we show that hPIPAC is possible in an ex-vivo model by combining and adapting medical devices available on the market.

## Methods

### Study design

This is an ex-vivo study in the inverted urinary bladder model (IBUB). All experiments were performed in triplicate.

### Regulatory background

Since no animals were used or sacrificed for the study, no local veterinary authority’s approval was needed.

### Occupational health safety

Distillate water was aerosolized instead of chemotherapy solutions to exclude any risk of exposition to toxic substances [17, 18].

### Inverted urinary bladder model (IBUB)

The IBUB model has been described elsewhere [19, 20]. The bovine bladder has a volume somewhat smaller (2–3 L) than the human abdominal cavity (3–5 L). Fresh organs were obtained from the slaughterhouse and immediately transported on ice ton the lab. A 4-cm incision was performed into the bladder neck. The bladder was inverted through the incision, which allows the exposition of the serosa on its inner side. A balloon trocar was inserted through the incision, and a ligature placed, ensuring full tightness. A second trocar was placed into the IBUB. The therapeutic capnoperitoneum was installed.

### Hyperthermic inverted urinary bladder model (hIBUB)

The IBUB model was adapted to allow experiments under hyperthermic conditions. Specifically, the hIBUB has to model the peritoneal membrane’s absorptivity, the thermal inertia of the human body, and the thermal conductivity through the peritoneum into the human body. Thermal inertia is defined as the degree of slowness with which the temperature of a body approaches its surroundings and depends upon its absorptivity, specific heat, thermal conductivity, dimensions, and other factors. Absorptivity is defined as the body’s property that determines the fraction of incident radiation absorbed by the body. Thermal conductivity is the quantity of heat that passes in unit time through a unit area of a plate whose thickness is unity when its opposite faces differ in temperature by one degree. The IBUB used in the experiments weighted between 109 and 128 g, and the target tissue volume to be heated was assumed to be equivalent to 110 ml water.

### Heating up dry and humid CO_2_

For generating hPIPAC, it was necessary to warm up CO_2_ in a closed environment saturated with H_2_O. Assumptions were adapted from Roth and Sea [21] and are summarized in Table 1.

**Table 1:** Assumptions for the physical parameters used in this study

Parameter	Value
CO_2_ insufflation pressure	775 mmHg^a^
Density of pure CO_2_	1.77 g/L^b^
Specific heat	0.206 cal/g/°C^b^
Density of water vapor	44.9 mg/L^b^
Heat of vaporization of water	580 cal/g^b^
Density of liquid water	0.998 g/mL
Specific heat of liquid water	1 cal/g/°C
Specific heat of human tissue	0.83 cal/g/°C ^c^
1 W	860 cal/h
1 cal	4184 J
Room temperature	20 °C
Normothermic conditions	37 °C
Hyperthermic conditions	41–43 °C, typically 42 °C
Initial weight of the inverted bovine urinary bladder (IBUB)	110 g

^a^15 mmHg above atmospheric pressure, assumed to be 760 mmHg

^b^At 37 °C and 775 mmHg

^c^Defined as the heat (in cal) required to raise the temperature of one gram of tissue by one degree Celsius

### Standard calorimetric calculation

We assumed that heat transfer from the IBUB lumen to the tissue would increase the peritoneal temperature using the standard calorimetric calculation

$$Q = m \times C \times \left( {T{\text{final}} - T{\text{initial}}} \right)$$

where *Q* is the heat flow, *m* the mass, *C* the specific heat, and *T* the temperature.

### Cooling effect of the gas

CO_2_ temperature at the output of the insufflator (Thermoflator, Karl Storz GmbH, Tuttlingen, Germany) was measured to be 33 °C. Insufflation of CO_2_ at 33 °C into the IBUB model under normothermic conditions (37 °C) has a cooling effect indeed. This cooling effect is proportional to the gas flow.

### Effect of humidity on heat transfer (normothermic conditions)

The amount of heat required to warm 1 L of dry CO_2_ from 33 °C to 37 °C = 1 L × 1.77 g/L × 0.206 cal/g/°C × 4 °C = 1.46 cal. Thus, it requires 146 cal to fully warm up 100 L dry CO_2_. However, this calculation does not yet reflect the abdomen's situation during laparoscopy, where the gaseous environment is saturated with humidity. When the dry CO_2_ enters the abdominal cavity, it is rapidly saturated with moisture, which has a cooling effect. Water has a high specific heat so that a significant amount of energy is needed to warm-up an environment saturated with humidity.

The amount of energy required to compensate for heat loss caused by the humid environment at 37 °C is 0.0449 g water/L × 580 cal/g per liter CO_2_ = 26.0 cal. Thus, 2600 cal is needed to thoroughly warm-up 100 L humid CO_2_. For hPIPAC calculations, the energy required to warm up the humid environment must be added to the energy needed to warm up dry CO_2_. The amount of heat necessary to warm up 100 L CO_2_ fully saturated (100%) with water is CO_2_ to 37 °C and 100% humidity = 146 + 2600 = 2746 cal (or 27.4 cal/L).

### Energy needed to establish intraluminal hyperthermic conditions

The calculations above are correct under normothermic conditions (37 °C). For generating intraluminal hyperthermic conditions (41–43 °C), additional heat has to be provided to the system.

### Energy needed to establish tissular hyperthermic conditions

The aim of hPIPAC is not to heat the intraluminal (humid) CO_2_, but to establish therapeutic hyperthermia in the target peritoneal tissue. There must indeed be an energy transfer from the warm-humid CO2 into this tissue for heating up peritoneal tissue. The peritoneum and subperitoneal tissue have a high water content, with a relative weight similar to the whole human body (specific gravity = 1.07). Thus, heating peritoneal tissue with intraluminal energy supply is a challenging task. The IBUB has an initial weight of around 110 g. From the above data, we can derive that the energy needed to heat the IBUB tissue from 37 to 42 °C is

$$Q \, = \, 110 {\text{g }} \times \, 0.83 {\text{cal}}/{\text{g}}/^\circ {\text{C }} \times \, \left( {42 ^\circ {\text{C }}{-} \, 37 ^\circ {\text{C}}} \right) \, = \, 456 \,{\text{cal}}$$

### Open or closed heat delivery system?

Humid (100% saturated) CO_2_ at 46 °C at a flow of 6 L/min carries 19.68 cal/min to the IBUB. We have calculated that 456 cal are needed to generate therapeutic hyperthermia. Thus, an estimate of 139 L CO_2_ over 23.17 min is necessary to deliver the energy required. Assuming a typical volume of an expanded IBUB between 2 and 3 L, the available gas volume is insufficient to transport the required amount of energy. Thus, an open heating system with continuous CO_2_ flow is necessary to provide enough energy to the target tissue.

### Cooling effect of the chemotherapy solution

The chemotherapy for PIPAC is diluted into a saline (NaCl 0.9%) or glucose 5% solution. Aerosolization of chemotherapy solutions in syringes at room temperature has a cooling effect on the tissue at body temperature. For simplification, we assumed that the specific heat of the chemotherapy solution to be equal to water. Thus, the amount of heat lost by aerosolizing 200 ml of crystalloid at 20 °C room temperature into the IBUB at 37 °C = 200 mL × 0.998 g/mL × 1 cal/g/°C × 17 °C = 3400 cal lost from the tissue into the CO_2_.

### Heat generation

The energy was provided to the system by the following means:

- CO_2_-insufflator: A thermic CO_2_ insufflation device was used (Thermoflator, Karl Storz GmbH, Tuttlingen, Germany). This device is CE-certified.
- A CO_2_ humidifying system (Humigard, Fisher & Paykel Healthcare, Auckland, New Zealand) was intercalated between the CO_2_ insufflator and the IBUB model. This system is CE-certified but had to be modified by the manufacturer to meet experimental demands.
- A heated tubing was connected between the surgical humidifying system and the IBUB (Humigard, Fisher & Paykel Healthcare, Auckland, New Zealand).
- Additional intraluminal energy was provided by a laparoscopic Saphir infrared coagulator (Licht-Koagulator, LC250, NK-Optik, München, Germany). This device is currently not CE-certified anymore.
- The syringes for chemotherapy solutions were placed into an angio-injector (Accutron HP-D, MedTron AG, Saarbrücken, Germany), keeping the pre-heated fluids at a constant temperature of 38–40 °C.

### Temperature*** measurements***

The temperature was measured as follows:

- Peritoneal tissue temperature: a digital probe (Thermohygrometer, Amarell, Kreuzwertheim, Germany) was sutured in a central position of the IBUB.
- Water bath temperature: A second, identical digital probe was immersed in the thermal bath.
- CO_2_ temperature: CO_2_ temperature was measured with an infrared laser thermometer (Infrarot Thermometer, ScanTemp385, Dostmann electronic GmbH, Mannheim, Germany) at two locations: a) at the exit of the CO_2_-insufflator and b) at the end of the warming tube.

### Data management

Data were collected manually and entered into an Excel file (Microsoft, Redmond, USA). For further analysis, data were transferred into a SPSS database v. 25 (IBM Inc, Armonk, NY, USA).

## Results

In the first step, the IBUB model was adapted to allow experiments taking into account thermal inertia, absorptivity, thermal conductivity, and thermal loss into the environment. For this purpose, inverted bovine urinary bladders (IBUB) were placed into a thermal bath to simulate heat loss from the (hyperthermic) peritoneal tissue into the (normothermic) human body caused by blood circulation. Since the water bath temperature was maintained at 37 °C by a thermostat, the thermal inertia of the thermal bath was considered infinite.

Then, a heating system consisting of three CE-certified elements (a CO_2_ insufflator, a surgical humidification system, and heated tubing) installed in this sequence was connected to the IBUB. Therapeutic hyperthermia (tissue temperature between 41 and 43 °C) was not reached in any experiments because of insufficient energy delivery to the target organ. The surgical humidification system's performance was boosted in the engineering laboratory to deliver the additional energy needed. Therapeutic hyperthermia in the target tissue could then be generated easily within 10–15 min (depending on the weight of the IBUB) by insufflating warm-humid CO_2_ at a flow of 6 L/min and a temperature of 46.8 °C. However, tissue temperature could not be maintained for more than a few minutes after the interruption of the CO_2_-flow. Since current standard operating procedures for PIPAC require an exposition period of 30 min with no gas flow (steady-state), this system was not sufficient for generating hPIPAC.

**Fig. 1:**
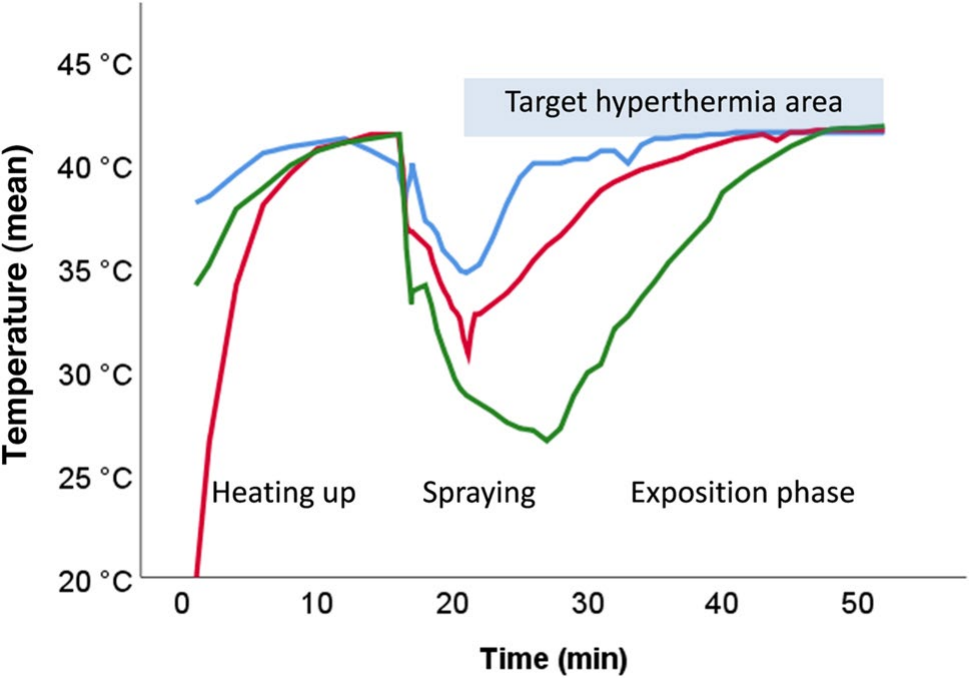
Target tissue temperature generated by warm-humid CO_2_ alone in the hyperthermic inverted bovine urinary bladder (hIBUB) model. Three phases are simulated: (1) heating the hIBUB with humid CO_2_ at a flow of 6 L/min and a temperature of 46.8 °C; (2) aerosolization of 200 ml chemotherapy at RT; (3) restoring therapeutic hyperthermia with warm-humid CO_2_. Warm-humid CO_2_ can generate therapeutic hyperthermia in the target tissue, but hyperthermia cannot be maintained during the aerosolization phase

As shown in Fig. 1, we simulated the thermic impact of aerosolizing 200 ml water at room temperature into the IBUB, and observed a dramatic fall in tissue temperature down to 31.1 ± 4.1 °C. This fall happened within 5 ± 1 min after starting the aerosolization despite the continuous flow of warm-humid CO_2_ (the Humigard was switched off but still attached). In the mean, 19 ± 5 min was needed to restore a target tissue temperature over 41 °C.

**Fig. 2:**
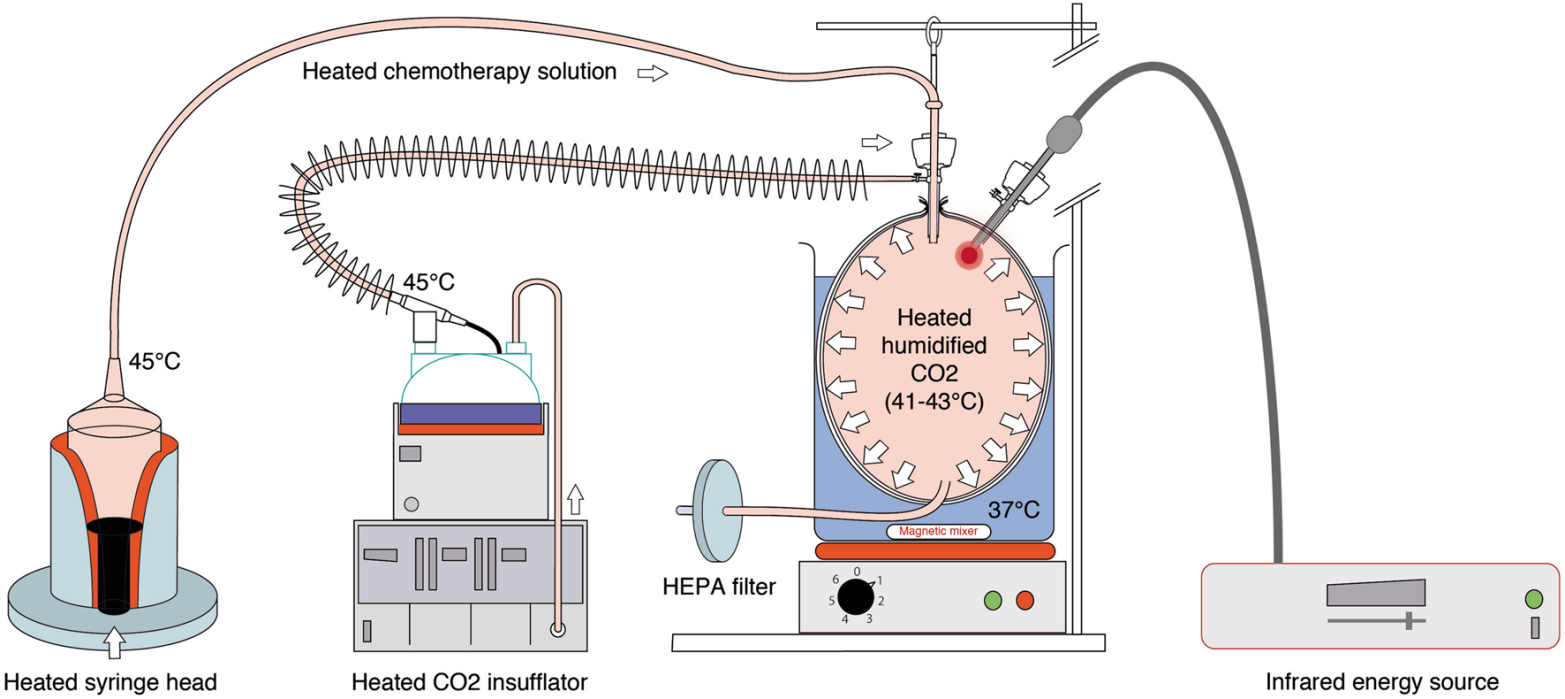
Technology proposed for generating hyperthermic pressurized intraperitoneal aerosol chemotherapy (hPIPAC). The system consists of the following components, connected sequentially: (a) an angio-injector equipped with a heating cuff; (b) a CO_2_-insufflator delivering dry CO_2_ at a temperature of 33 °C; (c) a device humidifying and warming up CO_2_ to an output temperature of 45 °C and (d) an endoscopic infrared Saphir coagulator inserted into the lumen of the hIBUB model

Thus, restoring therapeutic hyperthermia after aerosolization is relatively long and requires a continuous flow of warm-humid CO_2_. However, such continuous flow is prohibited by current PIPAC standards of practice. As shown in Fig. 2, two additional technical measures were taken to address this problem:

- The chemotherapy solution was pre-heated, and the syringes were placed into an angio-injector equipped with a heating cuff to prevent a fall in temperature during the aerosolization phase.
- An infrared coagulator was added to the system to provide additional intraluminal heat during the exposition (steady-state phase).

**Fig. 3:**
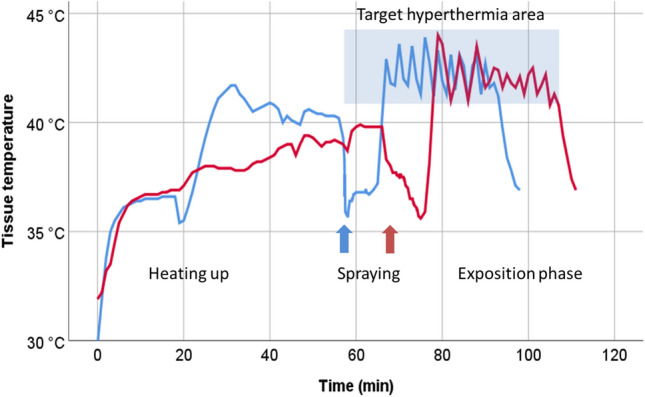
Target tissue temperature profile in two hIBUB organs (blue and red lines) during hPIPAC. The therapeutic phase begins at the end of the chemotherapy administration (spraying) phase. The infrared energy source restores and maintains the temperature of the target tissue within the desired therapeutic hyperthermia range (highlighted blue area) for the time needed (30 min) (Color figure online)

The integration of these new components was a success in generating and maintaining therapeutic hyperthermia in the target tissue, as shown in Fig. 3.

The function of the infrared source is illustrated in a video-clip recorded during the exposition phase (Additional material).

## Discussion

Hyperthermia (HIPEC) finds a broad acceptance in the surgical community as an adjunct to intraperitoneal chemotherapy for treating peritoneal metastasis. For 10 years, PIPAC is diffusing in clinical practice worldwide for treating patients who are no suitable candidates for CRS and HIPEC. Five years ago, H.H. Kim et al. proposed to combine PIPAC with hyperthermia, introducing the concept of hyperthermic PIPAC (H-PAC) [16]. However, to our knowledge, hPIPAC has not been used in clinical practice so far, probably because of the technological complexity and regulatory hurdles. In this work, we present a simple technology for generating hPIPAC and prove the feasibility of hPIPAC in an ex-vivo model.

Tumors located within a body cavity, such as peritoneal metastasis, are relatively easily accessible for interventional oncology procedures such as PIPAC. Thus, intracavitary heating techniques are a natural choice for generating hyperthermia for treating peritoneal metastasis. However, such an approach is challenging because intracavitary hyperthermia devices are impaired by their short (thermal) penetration depth [22].

Another challenge for generating hPIPAC is that the expanded abdomen is saturated with water during CO_2_ laparoscopy. Again, physical laws predict that it is extremely difficult or even impossible to warm up the target, diseased peritoneal tissue with heated, dry CO_2_. The temperature range of therapeutic hyperthermia lies between 41 and 43 °C. Using an advanced device with forced circulation of heated, dry CO_2_, the group of Kim generated intraperitoneal temperatures between 38.8 and 40.2 °C, which might appear relatively low [16]. Moreover, the technology infrastructure needed for generating hPIPAC seems expensive, complicated, and regulatory approval of this technology might be challenging.

Another group claimed that they developed an innovative technology (Hyperthermic intracavitary nanoaerosol therapy, HINAT) generating a nanometer-sized hyperthermic (41 °C) drug aerosol with a heatable liquid atomization unit (LAU) [23]. However, the authors report that “HINAT-LAU analyses were performed with an unheated aerosol” and provide no data on the temperature of the target tissue in their experiments. Thus, at the present point of time, the proof of concept of the ability of HINAT-LAU technology to generate and maintain therapeutic hyperthermia has not yet been delivered.

Building upon the reported experience of Kim co-author [16], our approach was to start with existing, certified industry-standard device, to combine and modify them in order to facilitate later clinical implementation. Moreover, we decided to warm-up humid CO_2_ instead of dry CO_2_ to increase energy transport and heat delivery into peritoneal tissue. Using this strategy, we demonstrated the feasibility of hPIPAC in an ex-vivo model, under experimental conditions close to clinical reality. However, this task was more challenging than expected, and combination of three devices (Humigard^®^, heating cuffs of the angioinjector and infrared device) was needed to ensure hyperthermic conditions over the time required (30 min). The beauty of PIPAC is the simplicity of the current set-up, and it is unclear if a more complex technology like hPIPAC is likely to be accepted by the clinical users. Whereas the use of CE-certified devices such as the Humigard^®^ device and the Chemo-HP^®^ angioinjector is possible in clinical practice, this is not the case for the infrared heating device yet. This infrared device was used in the 1990ies for liver hemostasis during laparoscopic liver resection. The device has lost its CE-certification in the meantime, and a new certification under the new European Medical Device Regulation is needed. This certification might be challenging to obtain due to potential risks (burns of intraabdominal organs) associated with this particular device.

Although we were technically successful in developing a relatively simple, effective, and affordable technology for hPIPAC, several challenges remain ahead. First, we have not demonstrated that hPIPAC has superior pharmacological properties as compared to PIPAC. Moreover, in-vivo experiments will be needed to confirm the biological advantages of hPIPAC versus other drug delivery techniques. Industrial and regulatory challenges have to be addressed before the first-in-human application. Then, controlled clinical trials will be needed to demonstrate the safety and efficacy of hPIPAC in human patients.

Even if such trials are successful, hPIPC will be required to document its superiority over existing or future comparators. hPIPAC is not the only research road to optimize the target tissue effect of intraperitoneal drug delivery. Other options are, for example, longer duration of exposition, increasing intraperitoneal pressure, ádvanced drug formulations, and electrostatic precipitation PIPAC (ePIPAC) [23–27].

In summary, we introduce a technology able to generate and maintain therapeutic hyperthermia between 41 and 43 °C in an ex-vivo model of peritoneal tissue. The technology proposed is relatively simple and might find a way to clinical application. The next steps are to investigate in functional models if hPIPAC offers pharmacological and/or biological benefits in the therapy of peritoneal metastasis, such as increased drug tissue concentration and penetration, tumor-selective cytotoxicity, increase immune response to cancer cells, or enhance the effect of chemotherapy.

## Supplementary Information

Below is the link to the electronic supplementary material.

Supplementary file1 (MP4 13396 kb)
